# Interrater Variability of ML-Based CT-FFR in Patients without Obstructive CAD before TAVR: Influence of Image Quality, Coronary Artery Calcifications, and Location of Measurement

**DOI:** 10.3390/jcm13175247

**Published:** 2024-09-04

**Authors:** Robin F. Gohmann, Adrian Schug, Christian Krieghoff, Patrick Seitz, Nicolas Majunke, Maria Buske, Fyn Kaiser, Sebastian Schaudt, Katharina Renatus, Steffen Desch, Sergey Leontyev, Thilo Noack, Philipp Kiefer, Konrad Pawelka, Christian Lücke, Ahmed Abdelhafez, Sebastian Ebel, Michael A. Borger, Holger Thiele, Christoph Panknin, Mohamed Abdel-Wahab, Matthias Horn, Matthias Gutberlet

**Affiliations:** 1 Department of Diagnostic and Interventional Radiology, Heart Center Leipzig, Strümpellstr. 39, 04289 Leipzig, Germany; adrian.schug@helios-gesundheit.de (A.S.); patrick.seitz@helios-gesundheit.de (P.S.); fyn.kaiser@helios-gesundheit.de (F.K.); sebastian.schaudt@helios-gesundheit.de (S.S.); katharina.renatus@helios-gesundheit.de (K.R.); paulkonrad.pawelka@helios-gesundheit.de (K.P.); sebastian.ebel@medizin.uni-leipzig.de (S.E.); matthias.gutberlet@helios-gesundheit.de (M.G.); 2 Medical Faculty, University of Leipzig, Liebigstr. 27, 04103 Leipzig, Germany; christian.krieghoff@helios-gesundheit.de (C.K.); christian.luecke@helios-gesundheit.de (C.L.); 3 Department of Cardiology, Heart Center Leipzig, University of Leipzig, Strümpellstr. 39, 04289 Leipzig, Germany; nicolas.majunke@medizin.uni-leipzig.de (N.M.); maria.buske@hmedizin.uni-leipzig.de (M.B.); steffen.desch@medizin.uni-leipzig.de (S.D.); ahmed.abdelhafez@medizin.uni-leipzig.de (A.A.); holger.thiele@medizin.uni-leipzig.de (H.T.); mohamed.abdelwahab@helios-gesundheit.de (M.A.-W.); 4 Department of Cardiac Surgery, Heart Center Leipzig, University of Leipzig, Strümpellstr. 39, 04289 Leipzig, Germany; sergey.leontyev@medizin.uni-leipzig.de (S.L.); thilo.noack@medizin.uni-leipzig.de (T.N.); philipp.kiefer@medizin.uni-leipzig.de (P.K.); michael.borger@medizin.uni-leipzig.de (M.A.B.); 5 Siemens Healthcare GmbH, Henkestr. 127, 91052 Erlangen, Germany; christoph.panknin@siemens-healthineers.com; 6 Institute for Medical Informatics, Statistics and Epidemiology (IMISE), University of Leipzig, Härtelstr. 16-18, 04107 Leipzig, Germany; matthias.horn@imise.uni-leipzig.de

**Keywords:** aortic stenosis, computed tomography coronary angiography, coronary angiography, coronary artery disease, transcatheter aortic valve implantation, diagnostic accuracy, machine learning

## Abstract

**Objectives***:* CT-derived fractional flow reserve (CT-FFR) can improve the specificity of coronary CT-angiography (cCTA) for ruling out relevant coronary artery disease (CAD) prior to transcatheter aortic valve replacement (TAVR). However, little is known about the reproducibility of CT-FFR and the influence of diffuse coronary artery calcifications or segment location. The objective was to assess the reliability of machine-learning (ML)-based CT-FFR prior to TAVR in patients without obstructive CAD and to assess the influence of image quality, coronary artery calcium score (CAC), and the location of measurement within the coronary tree. **Methods**: Patients assessed for TAVR, without obstructive CAD on cCTA were evaluated with ML-based CT-FFR by two observers with differing experience. Differences in absolute values and categorization into hemodynamically relevant CAD (CT-FFR ≤ 0.80) were compared. Results in regard to CAD were also compared against invasive coronary angiography. The influence of segment location, image quality, and CAC was evaluated. **Results**: Of the screened patients, 109/388 patients did not have obstructive CAD on cCTA and were included. The median (interquartile range) difference of CT-FFR values was −0.005 (−0.09 to 0.04) (*p* = 0.47). Differences were smaller with high values. Recategorizations were more frequent in distal segments. Diagnostic accuracy of CT-FFR between both observers was comparable (proximal: Δ0.2%; distal: Δ0.5%) but was lower in distal segments (proximal: 98.9%/99.1%; distal: 81.1%/81.6%). Image quality and CAC had no clinically relevant influence on CT-FFR. **Conclusions**: ML-based CT-FFR evaluation of proximal segments was more reliable. Distal segments with CT-FFR values close to the given threshold were prone to recategorization, even if absolute differences between observers were minimal and independent of image quality or CAC.

## 1. Introduction

Coronary computed tomography angiography (cCTA) is an excellent test for safely ruling out coronary artery disease [1] and is also employed in patients before transcatheter aortic valve replacement (TAVR) [2,3]. The diagnostic performance and specificity of cCTA can be improved by adding CT-derived fractional flow reserve (CT-FFR) to the diagnostic algorithm [4,5]. CT-FFR is a tool that in its original form is based on computational fluid dynamics (CFD) to simulate the blood flow in coronary vessels. CT-FFR combines this with the patient’s vessel anatomy derived from cCTA. This way, the blood flow can be computed at every point of the coronary tree in a resting state and simulated hyperemia. The result is a 3D, color-coded model of the coronary tree from which the CT-FFR values can be read [6]. In contrast to commercially available CFD-based CT-FFR, machine-learning (ML)-based CT-FFR can be performed on-site without time delay and potentially at a significantly lower cost. ML-based CT-FFR is based on a less computationally demanding algorithm and immediately renders results [7] based on a semi-automatic segmentation of the coronary arteries performed by physicians or other staff on site. ML-based CT-FFR correlates well with CFD CT-FFR [4] and may also improve diagnostic performance compared to cCTA in patients before TAVR [5,8,9,10,11,12].

CT-FFR is particularly useful for ruling out hemodynamically relevant coronary artery disease (CAD) in patients with intermediate stenoses on cCTA [13], as the latter is limited by its low specificity. Validation studies of CT-FFR have focused on CAD-positive (CAD^+^) patients and it is generally not recommended for patients without discrete stenoses (CAD^−^). A recent study on CAD^−^ patients before TAVR reported a very high count of false-positive ratings with especially distal CT-FFR values frequently being pathologically decreased [14]. A similar gradual decrease has also been described in invasive FFR in patients with diffuse atherosclerosis [15]. It is still unclear whether these distal measurements in CT-FFR reflect true pathophysiology in severe aortic stenosis (AS; e.g., imbalance of left ventricular myocardium and vessel cross section), diffuse arteriosclerosis, or may stem from a cumulative segmentation error. An analysis of interobserver correlation in CAD^−^ patients could help differentiate between inaccurate, random measurements or true pathophysiology and remains to be assessed [16,17,18]. While CT-FFR is not recommended in this patient cohort, the results of this scientific approach will contribute to better interpretate pathologically decreased distal values in the clinical setting. The chosen cohort of patients with severe AS allows the evaluation of a gradual CT-FFR decrease without the influence of significant CAD.

In this study, we analyzed the interrater variability of ML-based CT-FFR in patients without obstructive CAD before TAVR. Secondarily, we examined the influence of image quality, coronary artery calcium score (CAC), and the location of measurement.

## 2. Materials and Methods

### 2.1. Study Design and Patient Population

The patient population and study design have been described previously [14]. Consecutive CT examinations within a 7-month period of patients with severe aortic valve stenosis undergoing TAVR planning were included. Patients also received invasive coronary angiography (ICA) within 3 months of the CT. Of 388 patients, 116 showed no relevant stenosis (≥50%) on cCTA and were subsequently evaluated by two observers with CT-FFR.

The study was conducted in compliance with the Declaration of Helsinki (Medical Association 2013). The Ethics Committee of the University of Leipzig approved the study and written informed consent was waived (reference number: 435/18-ek).

### 2.2. CT Acquisition

The scan protocol has been described in a previous publication [3]. To reiterate, a non-enhanced scan for calcium scoring in standard technique was performed. This was followed by a single i.v. bolus of 70 mL iodinated contrast agent for a retrospectively ECG-gated helical CT scan of the heart and a non-gated high-pitch scan of the torso. No other medications were given. All examinations were performed on the same second-generation dual-source scanner (Somatom Definition Flash; Siemens, Erlangen, Germany).

### 2.3. Image Analysis

**Figure 1:**
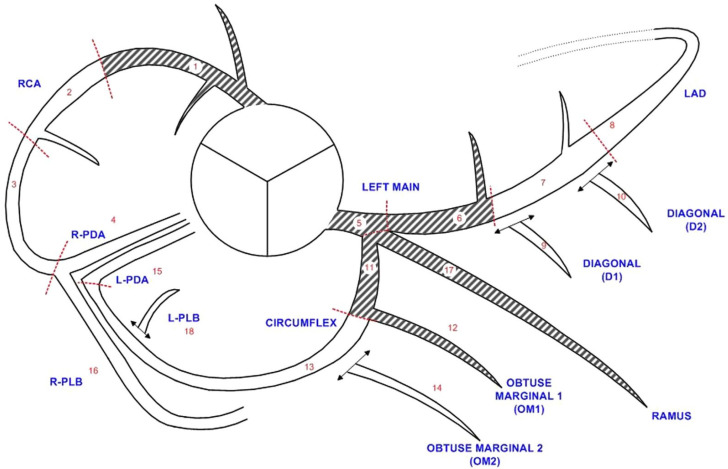
Diagram of the 18-segment model of the coronary tree. Diagram showing the coronary tree with its division into 18 segments according to the SCCT guidelines [16]. Shaded segments were defined as proximal in this study. L = left; LAD = left anterior descending artery; PDA = posterior descending artery; PLB = posterior-lateral branch; R = right; RCA = right coronary artery; SCCT = Society of Cardiovascular Computed Tomography.

The cCTA scans and ICA were analyzed by segment, based on the 18-segment model (Figure 1) [16]. For both cCTA and ICA with quantitative coronary angiography (QCA), a diameter stenosis of ≥50% was deemed relevant. ICA was used as the standard of reference in this study. Results on vessel and patient levels were formed by considering the respective worst segment (highest-grade stenosis). The assessment of qualitative and quantitative image quality has previously been described in detail [3,5]. Quantitative image quality consisted of contrast opacification and contrast to noise ratio, qualitative image quality categorized the examination as one of the following: 0 = nondiagnostic (excluded from this analysis, as CAD could not be excluded); 1 = diagnostic; 2 = good; 3 = excellent.

ML-based CT-FFR (cFFR version 3.2.0, Siemens, Erlangen, Germany; not commercially available) was performed on all patients without morphological signs of obstruction twice on the same examination by two observers. Observers A and B received the same instructions, while observer A had additionally received clinical training beforehand [17]. The CT-FFR prototype for on-site evaluation used in this study has been described extensively elsewhere [4,5,7].

Readings were taken at the junction point of the middle and distal third of each segment for vessels with a luminal diameter of >1.5 mm [14]. CT-FFR values ≤ 0.80 were considered to be indicative of hemodynamically relevant CAD [18]. Per-vessel and per-patient gradings were generated by considering the respective minimal CT-FFR value of the comprising segments. Gradings were compared between both observers and mismatches were classified as recategorizations. Evaluation time was measured from the time the dataset was loaded till the CT-FFR was computed, with the largest proportion of time allocated to centerline definition and segmentation of the vessel lumen.

For subsequent analyses, segments 1, 5, 6, 17, and 11 were arbitrarily considered proximal; all other segments were considered distal (Figure 1). The methods in this study comply with the Guidelines for Reporting Reliability and Agreement Studies (GRRAS) [19].

### 2.4. Statistics

Body mass index and age were given as mean ± standard deviation. Asymmetric distributions for evaluation times and pairwise interobserver differences were given as median and interquartile range (IQR). In addition, 95% confidence intervals (CIs) of the median differences were specified. To assess differences in median CT-FFR values of the observers, a Wilcoxon signed-rank test was performed. Interrater agreement was calculated using the intra-class correlation coefficient (ICC) of type ICC (3,1) [20] with the corresponding 95% CI. For the interpretation of ICC values, Cicchetti’s guidelines were employed [21] wherein values less than 0.4 and between 0.4 and 0.6 were considered indicative of poor and fair correlation, respectively. Values between 0.6 and 0.75 suggested good agreement; values exceeding 0.75 signified excellent correlation.

To determine the correlation between CT-FFR differences and covariates, Spearman’s rank correlation (quantitative image quality measures and CAC) or Kendall’s rank correlation (qualitative image quality) were calculated. The correlation of CAD categorization between both observers and covariates was assessed using point-biserial correlation (quantitative image quality and calcium burden) or rank-biserial correlation (qualitative image quality). *p*-values of all correlation analyses are with respect to the null hypothesis that the correlation coefficient equals zero. *p*-values < 0.05 were considered statistically significant.

Data curation and computation of inferential statistics were performed with spreadsheets (Microsoft Excel version 2010, Microsoft Corporation, Redmond, WA, USA). For further statistical analyses, R (v4.3.3, R Foundation for Statistical Computing, Vienna, Austria) was used.

## 3. Results

### 3.1. Study Population

In this study, 109 of 116 CT examinations of CAD^−^ patients were successfully evaluated by both observers. Initial exclusions occurred because of borderline image quality of cCTA rendering segmentation for CT-FFR not feasible (n = 3) and anatomical variants outside of the model boundaries of the CT-FFR prototype (n = 4) [14]. No additional exclusions occurred. The included patients had a mean body mass index of 28.4 ± 5.5 kg/m^2^, a mean age of 78.4 ± 7.2 years, and 64.2% were female [3]. The median evaluation time of CT-FFR was 21 (16–28) min. and 24 (19–29) min. for observers A and B, respectively.

### 3.2. Absolute Differences

**Figure 2:**
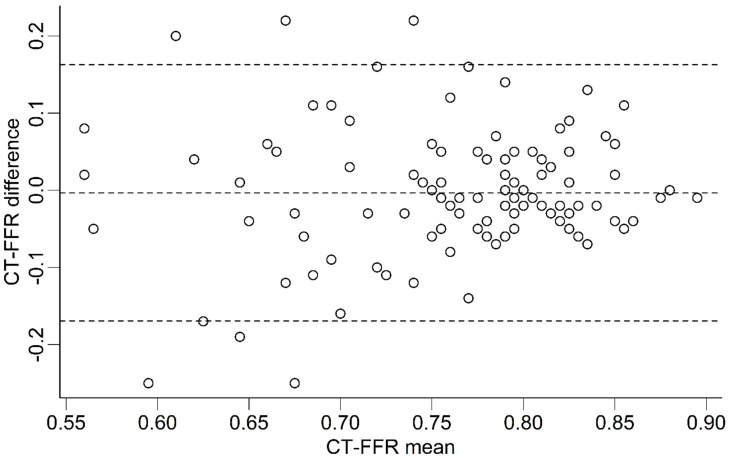
CT-FFR values at patient level. Bland-Altman plot showing the distribution of differences in CT-FFR values between both observers. Differences between both observers are overall very small. The plot shows no indication of a systematic bias. Lower mean CT-FFR values show one outlier (CT-FFR difference of −0.45 at a CT-FFR mean value of 0.58), which has been omitted from the plot for clarity. Lower mean CT-FFR values also show a larger heterogeneity of CT-FFR differences. CT-FFR = CT-derived fractional flow reserve. Dashed horizontal lines represent the mean difference (middle line) and the upper and lower limits of agreement (±1.96 SD, upper and lower lines).

Absolute CT-FFR values were very similar between the observers (Figure 2) and showed no significant median difference on patient (n = 109; −0.005 [−0.09 to 0.04]; *p* = 0.47) or vessel level (*p* ≥ 0.12; Table 1). On segment level, median differences were also very low, with few of these differences reaching statistical significance (Table 1). Between proximal and distal segments, there was no discernable trend toward higher or lower differences in CT-FFR values. All data is shown in Table 1.

### 3.3. Interobserver Variability

Analysis on patient level showed a fair correlation of absolute CT-FFR values between both observers (ICC = 0.421; *p* < 0.001). The LAD showed a poor correlation (ICC = 0.334; *p* < 0.001); the other vessels showed fair (LM: ICC = 0.507; *p* < 0.001; LCX: ICC = 0.588; *p* < 0.001) or good agreement (RCA: ICC = 0.701; *p* < 0.001) of CT-FFR values. The segments forming the LAD showed mostly a poor correlation. Segments forming the other vessels showed a better correlation (RCA > LM > LCX; Table 1). No trend towards higher or lower correlation in distal segments was found.

Between both observers, recategorizations into hemodynamically relevant CAD occurred in 29.4% of patients. On vessel level, the LAD showed the highest number of recategorizations, whereas the LM showed the lowest (LAD: 31.2% > RCA: 26.6% > LCX: 20.2% > LM: 0%) (Table 1). Recategorizations in proximal segments (median: 0.9%) were less frequent than in distal segments (median: 14.8%).

### 3.4. Diagnostic Performance

Diagnostic performance on patient level was identical between both observers (sensitivity: 100%; specificity: 29.0%; diagnostic accuracy: 30.3%; Table 2). On vessel level, observer B showed slightly better specificity and accuracy of Δ +1.8% and Δ +2.1%, respectively. The specificity and diagnostic accuracy of observer B were minimally higher for segmental measurements (Δ +0.4%). All values can be found in Table 2.

### 3.5. Localization of Measurement

Proximal segments showed higher diagnostic accuracy and specificity than distal segments for both observers (accuracy: 98.9%/99.1% vs. 81.1%/81.6%; specificity: 98.9%/99.1% vs. 81.3%/81.8%; Table 3). Differences between both observers were very small with slightly smaller differences in proximal segments compared to distal ones (accuracy and specificity: Δ −0.2% vs. Δ −0.5%). Further detail is shown in Table 3.

### 3.6. Influence of Image Quality and Coronary Artery Calcifications

On patient level, absolute and categorized CT-FFR values did not correlate with any image quality parameters. On vessel level, there were weak correlations between absolute CT-FFR values and contrast opacification in the LM (r = 0.204; *p* = 0.03) and between categorized values and contrast opacification in the RCA (r = 0.218; *p* = 0.02). Coronary artery calcium burden correlated weakly with absolute CT-FFR values on patient level (r = 0.394; *p* < 0.001) and in the LAD (r = 0.393; *p* < 0.001). Categorized CT-FFR values only showed a weak correlation with CAC in the LCX (r = 0.207; *p* = 0.03). Further detail is given in Table 4.

## 4. Discussion

Overall, CT-FFR values of patients before TAVR without obstructive CAD on cCTA were highly consistent between the observers with only sporadic differences on segment level. Despite these small differences and identical diagnostic performance on patient level, numerous recategorizations into hemodynamically relevant CAD occurred, and correlation according to CT-FFR values ranged from good to even poor in several instances. Interestingly, differences in categorization were much smaller in proximal coronary artery segments and were generally independent of image quality and CAC.

Absolute CT-FFR values showed very small differences between both observers (Figure 2). In comparison to other studies on patients with obstructive CAD, our average differences on patient level were roughly one magnitude smaller (our results: −0.005 (−0.09 to 0.04), *p* = 0.47; CAD^+^ patients before TAVR: −0.05 (−0.12 to 0.02); *p* < 0.001 [17]; Gaur et al.: 0.011 ± 0.034 [22]). The presence of CAD appears to be associated with an increase in variability, even more so in patients with multiple comorbidities. Gaur et al. were the first to publish data on the variation of values determined by the commercially available CFD CT-FFR algorithm [22]. Differences were higher than in this CAD^−^ cohort, but similar to those of invasive FFR measurements. However, the details of the segmentation process or the observer experience remain undisclosed.

The sporadic statistically significant differences found in several segments should not be over-interpreted in light of the only weak associations found and the sheer number of tests performed. A possible explanation for the small differences may be that CT-FFR segmentation for computation of CT-FFR in CAD^−^ patients could rely more heavily on the semi-automatic segmentation algorithm and is thus less susceptible to variation by manual intervention, e.g., correction of the luminal contours at calcified plaques. Interestingly, the interrater agreement of absolute CT-FFR values on patient level in comparison to our previous publication is lower than the much smaller absolute differences may lead to expect (our results: ICC = 0.421, *p* < 0.001; CAD^+^ patients: ICC = 0.567, *p* < 0.001) [17]. One possible reason for this is the relatively homogenous data set compared to CAD^+^ patients with reduced variability having the effect of potentially decreasing parameters like the ICC. Other studies thus showed higher ICC values, while examining different patient cohorts with also generally more experienced observers [8,23,24,25]. Less experienced observers typically achieve lower correlations in CT-FFR [26,27] as well as in the morphological assessment of cCTA and ICA [28,29,30]. However, in light of the low absolute differences, we do not believe this to be the primary explanation for the only moderate correlation of CT-FFR values, as supported by the reasons mentioned above [25,26]. Still, standardized training of observers is likely critical to secure robustness and reliability. Therefore, clinical application of ML-based CT-FFR should be carried out by experienced staff.

Evaluation on vessel level showed that the correlation of values in the LAD was the lowest. A possible explanation is that the LAD was the vessel with the most included segments in our cohort (mean: 4.6 segments/patient) thus entailing the highest potential for cumulative segmentation error. Additionally, segments 9, 10, and 17 (LAD side branches) with their respective bifurcation increase the difficulty of the segmentation process enabling potential errors in assignment.

Differences in categorization into hemodynamically relevant CAD between both observers occurred frequently, without affecting diagnostic accuracy on patient level at all. Considering these findings alongside the exceedingly small absolute median differences, it is evident that many reclassified segments likely possess CT-FFR values in close proximity to the threshold, thereby increasing the likelihood of ‘threshold jumps’ due to the minimal discrepancies observed during segmentation. A lower diagnostic power of CT-FFR values was described around the cut-off of ≤0.80 [31]. These values also correlated less well with invasive FFR in another study [32]. This study highlights that dichotomous decision-making should be discouraged with CT-FFR values around the cut-off (particularly values between 0.76 and 0.80), and additional risk stratification is needed before a treatment decision is made to account for the increased variability [13].

In our study, recategorizations were lower in proximal segments compared to distal ones (median: 0.9% vs. 14.8%). Thus, the assessment of proximal segments seems more reliable. The increasing number of recategorizations distally is most likely the result of a cumulative segmentation error. As this may alter CT-FFR values in either direction, threshold jumps for CT-FFR values close to the cut-off become more probable. Because this study was performed on CAD^−^ patients, the gradually decreasing values along any vessel occurred independently of discrete coronary artery stenoses. Generally, a decrease of 0.08 to 0.13 has been reported in the literature for CAD^+^ patients too [33]. The approach of this study enables the assessment of this phenomenon without the influence of stenoses. It can be assumed that these hemodynamic changes also occur in CAD^+^ patients. Similarly, Tsugu et al. recently reported that the lumen volume-to-vessel-length ratio was the strongest predictor after calcified plaque volume for CT-FFR ≤ 0.80 in the RCA in patients with non-obstructive CAD [34]. Additionally measuring the volume/length ratio might improve diagnostic performance distally for all patients undergoing CT-FFR in the future. Our study cohort before TAVR may present a special case because of severe AS with ultimately altered hemodynamics and frequent coinciding left ventricular myocardial hypertrophy and diffuse atherosclerosis. These may lead to an imbalance between vessel cross-section and muscle mass and thereby alter CT-FFR values [33].

To evaluate distal segments more reliably, changing the cut-off may be beneficial. The currently employed cut-off of ≤0.80 was proposed after evaluating lesions independent of their location [13,35]. A more lenient cut-off distally could factor in the gradual CT-FFR decrease and reduce the number of false positive ratings. Another way to mitigate this issue could be to apply ΔCT-FFR between two or more points of measurement along a vessel, rather than an absolute cut-off. An abrupt pressure drop along the coronary vessel has high diagnostic accuracy for relevant CAD [13,36,37]. Furthermore, in a recent study, Chen et al. found changes in CT-FFR values potentially useful for monitoring treatment efficacy in diffuse atherosclerosis [38].

The diagnostic performance of both observers was identical on patient level, with only minimal variances observed at vessel and segment levels. Looking at the high rate of recategorizations, especially on patient level, equal numbers of recategorizations from true to false and vice versa between both observers occurred (Table 1 and Table 2; ∆accuracy: 0.0%; recategorizations: 29.4%). This reinforces the assumption that recategorizations are more likely caused by a random error affecting both, rather than, e.g., one observer’s subpar/worse segmentation. As the prevalence of CAD is very low in our study cohort (n = 2), no meaningful findings could be generated concerning PPV, NPV, and absolute values of diagnostic accuracy. Hence, the values are listed to compare observers and should not be compared to other patient populations.

In proximal segments, higher diagnostic accuracy and specificity were observed (Table 3). This is analogous to the lower rate of recategorization in those segments discussed above and points towards a cumulative segmentation error with increasing importance distally. Conversely, the ICC showed no trend towards lower values distally. However, any trend could be masked by high ICC fluctuations due to the statistical impact of small differences within the otherwise homogenous data set. Similarly, Renker et al. reported in a CAD^+^ patient cohort lower lesion-specific diagnostic accuracy in distal segments with ML-based CT-FFR compared to invasive FFR serving as the standard of reference. Nonetheless, ML-based CT-FFR remained superior to cCTA alone [39] and is aimed at widespread clinical application. Current guidelines today already recommend (CFD) CT-FFR after cCTA evaluation for patients with intermediate pretest probability for CAD with intermediate stenosis [40]. Issues like relatively long evaluation time could be mitigated by further improving patient selection and image quality, e.g., with third-generation dual-source CTs.

Only sporadically, weak correlations between image quality or CAC burden and CT-FFR were found. The few sporadic significant correlations that were found in either absolute CT-FFR values or categorized values did not seem to be connected. Several other studies have published similar results [17,23,24,35]. On the other hand, for obvious reasons, a very high calcium burden may influence the readability of cCTA and ultimately CT-FFR [41]. Image quality parameters had no clinically meaningful influence on the consistency of CT-FFR values. A possible explanation for this may be that once image quality is sufficient to exclude relevant CAD reliably, neither image quality nor CAC burden significantly interferes with confident lumen segmentation.

Values obtained in proximally located segments appear more reliable. The gradual decrease of CT-FFR values distally leads to values falling closer to the cut-off of ≤0.80, making threshold jumps with the coinciding cumulative segmentation error more probable. Image quality and CAC seem not to have a major influence. A way to mitigate this would be the clinical application of a location-dependent threshold or ∆CT-FFR along a stenosis. Further studies of CT-FFR with correlation to invasive tests (e.g., invasive FFR) are needed. Lastly, an interesting subject for further studies is how reliable CT-FFR is on CAD^−^ patient cohorts without severe AS, as AS might facilitate low CT-FFR values.

### Limitations

Several important limitations of this study must be mentioned. The cCTA data were acquired without the use of nitroglycerin or beta-blockers, likely adding to the challenges of interpretation, rather than artificially enhancing the results. Also, this study compared functional CT-FFR values with a morphological standard of reference (QCA ≥ 50%). A functional standard of reference would have been desirable to have more certainty of actual possible hemodynamic changes in patients before TAVR. These patients have severe AS and often have myocardial hypertrophy and comorbidities such as CAD. The aforementioned myocardial hypertrophy being relevant for the computation of CT-FFR is potentially contributing to the gradually decreasing CT-FFR values in our study cohort. As this was a single-center study employing a not commercially available prototype outside of its aimed for CAD^+^ patient cohort, the generalizability of our findings must be conducted cautiously.

Furthermore, both observers had a moderate amount of experience with the method beforehand, whereas other studies with better correlation were often carried out by experts with several years of clinical experience [24]. The comparability to the study on CFD CT-FFR variability is limited because a very small CAD^+^ patient cohort (n = 28) was assessed and their segmentation process remained undisclosed [22].

## 5. Conclusions

Differences in absolute CT-FFR values in CAD^−^ patients were very small, enabling a reliable evaluation of proximal segments. Nevertheless, a gradual decrease in CT-FFR value towards distal segments is known and in combination with a small coinciding cumulative segmentation error leads to a substantial amount of threshold jumps and consequently recategorizations, particularly in distal segments. Interobserver variability was independent of image quality and CAC, suggesting these abnormal measurements to be related to pathophysiological changes in severe AS. Though not useful for its original indication, these results are encouraging the use of CT-FFR also in challenging patient groups, potentially enabling novel uses, e.g., monitoring of diffuse CAD.

## Figures and Tables

**Table 1:** Interobserver variability of absolute CT-FFR values.

Level of Observation		n	Difference	95% CI	*p*	ICC	95% CI	*p*	RoR %	95% CI
**Patient**		109	−0.005 (−0.09 to 0.04)	−0.02, 0.01	0.47	0.421	0.25, 0.56	<0.001	29.4	21.6, 38.5
**Vessel**	**RCA**	109	0.0 (−0.03 to 0.05)	−0.01, 0.02	0.62	0.701	0.59, 0.79	<0.001	26.6	19.2, 35.6
	**LM**	109	0.0 (0.00 to 0.01)	0.00, 0.01	0.14	0.507	0.35, 0.63	<0.001	0.0	0.0, 3.4
	**LAD**	109	−0.015 (−0.08 to 0.04)	−0.03, 0.01	0.12	0.334	0.16, 0.49	<0.001	31.2	23.3, 40.4
	**LCX**	109	0.00 (−0.05 to 0.05)	−0.02, 0.01	0.76	0.588	0.45, 0.70	<0.001	20.2	13.7, 28.7
**Segments**	**S1**	109	0.01 (−0.01 to 0.01)	0.01, 0.02	<0.001	0.498	0.34, 0.63	<0.001	0.0	0.0, 3.4
	**S2**	108	0.015 (−0.01 to 0.03)	0.01, 0.02	<0.001	0.725	0.62, 0.80	<0.001	1.9	0.5, 6.5
	**S3**	101	0.015 (−0.02 to 0.04)	0.01, 0.03	0.003	0.724	0.62, 0.81	<0.001	13.9	8.4, 21.9
	**S4**	76	−0.005 (−0.04 to 0.03)	−0.02, 0.01	0.46	0.73	0.61, 0.82	<0.001	28.9	20.0, 40.0
	**S16**	80	0.00 (−0.04 to 0.04)	−0.01, 0.02	0.83	0.676	0.54, 0.78	<0.001	21.2	13.7, 31.4
	**S5**	109	0.00 (0.00 to 0.01)	0.00, 0.01	0.14	0.507	0.35, 0.63	<0.001	0.0	0.0, 3.4
	**S6**	109	0.015 (−0.01 to 0.02)	0.01, 0.02	<0.001	0.432	0.27, 0.57	<0.001	0.9	0.2, 5.0
	**S7**	109	0.00 (−0.05 to 0.03)	−0.02, 0.01	0.81	0.349	0.17, 0.50	<0.001	9.2	5.1, 16.1
	**S8**	108	−0.01 (−0.08 to 0.03)	−0.03, 0.01	0.17	0.362	0.19, 0.52	<0.001	29.6	21.8, 38.8
	**S9**	88	0.00 (−0.05 to 0.04)	−0.02, 0.02	0.77	0.343	0.15, 0.52	<0.001	14.8	8.8, 23.7
	**S10**	56	−0.01 (−0.06 to 0.03)	−0.04, 0.01	0.42	0.475	0.24, 0.65	<0.001	17.9	10.0, 29.8
	**S17**	34	0.01 (−0.03 to 0.03)	−0.02, 0.04	0.35	0.304	−0.03, 0.58	0.04	14.7	6.4, 30.1
	**S11**	109	0.01 (−0.01 to 0.03)	0.01, 0.02	<0.001	0.485	0.33, 0.62	<0.001	2.8	0.9, 7.8
	**S12**	88	0.005 (−0.03 to 0.04)	−0.01, 0.02	0.38	0.297	0.10, 0.48	0.002	10.2	5.5, 18.3
	**S13**	90	0.015 (−0.02 to 0.05)	0.01, 0.03	0.009	0.554	0.39, 0.68	<0.001	11.1	6.1, 19.3
	**S14**	58	0.00 (−0.06 to 0.04)	−0.02, 0.02	1.00	0.621	0.43, 0.76	<0.001	19.0	10.9, 30.9
	**S15**	11	0.005 (−0.03 to 0.06)	−0.06, 0.07	0.89	0.619	0.07, 0.88	0.02	9.1	1.6, 37.7
	**S18**	13	−0.024 (−0.06 to 0.06)	−0.09, 0.03	0.33	0.430	−0.13, 0.78	0.06	30.8	12.7, 57.6

Values are median difference (and IQR) of CT-FFR values, the intra-class correlation coefficient (ICC), and rate of recategorizations of the two observers. The first *p*-value column (and 95% CI) corresponds to the difference of median CT-FFR differences from zero while the second *p*-value column (and 95% CI) corresponds to the difference of the ICC from zero. *p*-values < 0.05 were statistically significant. CI = confidence interval; ICC = intra-class correlation coefficient; IQR = interquartile range; LAD = left anterior descending artery; LM = left main coronary artery; LCX = circumflex artery; RoR = rate of recategorization; RCA = right coronary artery.

**Table 2:** Comparison of diagnostic performance of observer A and B.

Level of Observation	Observer	n	TP	TN	FP	FN	Sen. %	Spe. %	PPV %	NPV %	Acc. %
**Patient**	**Observer A**	109	2	31	76	0	100.0%	29.0	2.6	100.0	30.3
	**Observer B**		2	31	76	0	100.0%	29.0	2.6	100.0	30.3
	**Difference Δ**		0	0	0	0	0.0%	0.0	0.0	0.00	0.0
**Vessel**	**Observer A**	436	0	306	128	2	0.0%	70.5	0.0	99.4	70.2
	**Observer B**		1	314	120	1	50.0%	72.4	0.8	99.7	72.3
	**Difference Δ**		1	8	−8	−1	50.0%	+1.8	+0.8	+0.3	+2.1
**Segment**	**Observer A**	1456	0	1265	189	2	0.0%	87.0	0.0	99.8	86.9
	**Observer B**		0	1271	183	2	0.0%	87.4	0.0	99.8	87.3
	**Difference Δ**		0	6	−6	0	0.0%	+0.4	0.0	0.0	+0.4

ICA was used as the standard of reference. Thresholds for coronary artery disease were ICA ≥ 50% diameter and CT-FFR ≤ 0.80. Acc. = accuracy; CT-FFR = CT-derived fractional flow reserve; FN = false negative; FP = false positive; ICA = invasive coronary angiography; NPV = negative predictive value; PPV = positive predictive value; Sen. = sensitivity; Spe. = specificity; TN = true negative; TP= true positive.

**Table 3:** Comparison of diagnostic performance of observers A and B depending on segment location.

Segment Location	Observer	n	TP	TN	FP	FN	Sen. %	Spe. %	PPV %	NPV %	Acc. %
**Proximal**	**Observer A**	470	0	465	5	0	/	98.9%	0.0%	100.0%	98.9%
	**Observer B**		0	466	4	0	/	99.1%	0.0%	100.0%	99.1%
	**Difference Δ**		0	−1	1	0	/	−0.2%	0.0%	0.0%	−0.2%
**Distal**	**Observer A**	986	0	800	184	2	0.0%	81.3%	0.0%	99.8%	81.1%
	**Observer B**		0	805	179	2	0.0%	81.8%	0.0%	99.8%	81.6%
	**Difference Δ**		0	−5	5	0	0.0%	−0.5%	0.0%	0.0%	−0.5%

ICA was used as the standard of reference. Thresholds for coronary artery disease were ICA ≥ 50% diameter and CT-FFR ≤ 0.80. Acc. = accuracy; CT-FFR= CT-derived fractional flow reserve; FN = false negative; FP = false positive; ICA = invasive coronary angiography; NPV = negative predictive value; PPV = positive predictive value; Sen. = sensitivity; Spe. = specificity; TN = true negative; TP = true positive.

**Table 4:** Influence of image quality and coronary arterial calcifications on recategorization.

CT-FFR Values	Level of Observation		n	Image Quality									Calcium Burden		
				r CNR	95% CI	*p*	r HU	95% CI	*p*	r QIQ	95% CI	*p*	r CAC	95% CI	*p*
**Absolute**	**Patient**		109	−0.059	−0.241, 0.127	0.55	−0.073	−0.261, 0.120	0.45	−0.064	−0.208, 0.080	0.40	0.394	0.221, 0.542	<0.001
	**Vessel**	**RCA**	109	−0.032	−0.223, 0.157	0.74	−0.081	−0.276, 0.121	0.41	−0.127	−0.283, 0.033	0.10	0.078	−0.115, 0.264	0.42
		**LM**	109	0.173	−0.038, 0.363	0.07	0.204	−0.010, 0.396	0.03	0.004	−0.178, 0.184	0.97	0.001	−0.184, 0.192	1.00
		**LAD**	109	−0.122	−0.311, 0.075	0.21	−0.146	−0.340, 0.059	0.13	−0.094	−0.246, 0.066	0.22	0.393	0.200, 0.553	<0.001
		**LCX**	109	0.090	−0.102, 0.276	0.35	0.066	−0.135, 0.260	0.50	−0.026	−0.175, 0.128	0.73	0.129	−0.069, 0.311	0.18
**Categorized**	**Patient**		109	0.011	−0.177, 0.199	0.91	−0.040	−0.226, 0.149	0.68	0.009	−0.227, 0.243	0.94	0.133	−0.057, 0.313	0.17
	**Vessel**	**RCA**	109	0.114	−0.076, 0.296	0.24	0.218	0.031, 0.390	0.02	−0.226	−0.444, 0.016	0.05	0.013	−0.176, 0.200	0.90
		**LAD**	109	0.118	−0.071, 0.300	0.22	0.091	−0.098, 0.275	0.35	−0.019	−0.249, 0.213	0.86	0.099	−0.091, 0.282	0.31
		**LCX**	109	−0.020	−0.207, 0.169	0.84	-0.061	−0.246, 0.129	0.53	0.139	−0.131, 0.389	0.28	0.207	0.019, 0.380	0.03

Correlation of CNR, contrast opacification (measured in HU), qualitative image quality, or CAC with either absolute CT-FFR values or categorization into hemodynamically relevant CAD. The first *p*-value column (and 95% CI) corresponds to CNR, the second to HU, and the third to QIQ, while the fourth *p*-value column (and 95% CI) corresponds to CAC. All *p*-values are with respect to the null hypothesis that no correlation exists (correlation coefficient r = 0). *p*-values < 0.05 were considered statistically significant. Thresholds for significant CAD were ≥50% diameter for QCA and ≤0.80 for CT-FFR, respectively. The LM was excluded in categorized values because no recategorizations were observed in the cohort. CAC = coronary artery calcium score, CAD = coronary artery disease, CI = confidence interval; CNR = contrast-to-noise ratio; CT-FFR = CT-derived fractional flow reserve; HU = Hounsfield units; LAD = left anterior descending artery; LM = left main coronary artery; LCX = circumflex artery; QCA = quantitative coronary angiography; QIQ = qualitative image quality; RCA = right coronary artery.

## Data Availability

The datasets generated and/or analyzed during the current study are not publicly available due to German Data Protection laws but are available from the corresponding author upon reasonable request after approval of the local ethics committee and data safety board.

## Abbreviations

AS	aortic stenosis
CAC	coronary artery calcium score
CAD	coronary artery disease
CAD^+^	positive for coronary artery disease
CAD^−^	negative for coronary artery disease
cCTA	coronary CT-angiography
CFD	computational fluid dynamics
CT-FFR	CT-derived fractional flow reserve
ICA	invasive coronary angiography
ICC	intra-class correlation coefficient
ML	machine learning
LM	left main coronary artery
LAD	left anterior descending coronary artery
LCX	circumflex coronary artery
QCA	quantitative coronary angiography
RCA	right coronary artery
TAVR	transcatheter aortic valve replacement

## References

[1] Maurovich-Horvat P., Bosserdt M., Kofoed K.F., Rieckmann N., Benedek T., Donnelly P., Rodriguez-Palomares J., Erglis A., Štěchovský C., Šakalyte G. (2022). CT or Invasive Coronary Angiography in Stable Chest Pain. N. Engl. J. Med..

[2] van den Boogert T.P.W., Vendrik J., Claessen B.E.P.M., Baan J., Beijk M.A., Limpens J., Boekholdt S.A.M., Hoek R., Planken R.N., Henriques J.P. (2018). CTCA for Detection of Significant Coronary Artery Disease in Routine TAVI Work-up: A Systematic Review and Meta-Analysis. Neth. Heart J..

[3] Gohmann R.F., Lauten P., Seitz P., Krieghoff C., Lücke C., Gottschling S., Mende M., Weiß S., Wilde J., Kiefer P. (2020). Combined Coronary CT-Angiography and TAVI-Planning: A Contrast-Neutral Routine Approach for Ruling-Out Significant Coronary Artery Disease. J. Clin. Med..

[4] Coenen A., Kim Y.-H., Kruk M., Tesche C., De Geer J., Kurata A., Lubbers M.L., Daemen J., Itu L., Rapaka S. (2018). Diagnostic Accuracy of a Machine-Learning Approach to Coronary Computed Tomographic Angiography–Based Fractional Flow Reserve. Circ. Cardiovasc. Imaging.

[5] Gohmann R.F., Pawelka K., Seitz P., Majunke N., Heiser L., Renatus K., Desch S., Lauten P., Holzhey D., Noack T. (2022). Combined CCTA and TAVR Planning for Ruling Out Significant CAD: Added Value of ML-Based CT-FFR. JACC Cardiovasc. Imaging.

[6] Taylor C.A., Fonte T.A., Min J.K. (2013). Computational Fluid Dynamics Applied to Cardiac Computed Tomography for Noninvasive Quantification of Fractional Flow Reserve: Scientific Basis. J. Am. Coll. Cardiol..

[7] Itu L., Rapaka S., Passerini T., Georgescu B., Schwemmer C., Schoebinger M., Flohr T., Sharma P., Comaniciu D. (2016). A Machine-Learning Approach for Computation of Fractional Flow Reserve from Coronary Computed Tomography. J. Appl. Physiol..

[8] Langenbach M.C., Langenbach I.L., Foldyna B., Mauri V., Klein K., Macherey-Meyer S., Heyne S., Meertens M., Lee S., Baldus S. (2024). Advanced CT Measures of Coronary Artery Disease with Intermediate Stenosis in Patients with Severe Aortic Valve Stenosis. Eur. Radiol..

[9] Brandt V., Schoepf U.J., Aquino G.J., Bekeredjian R., Varga-Szemes A., Emrich T., Bayer R.R., Schwarz F., Kroencke T.J., Tesche C. (2022). Impact of Machine-Learning-Based Coronary Computed Tomography Angiography-Derived Fractional Flow Reserve on Decision-Making in Patients with Severe Aortic Stenosis Undergoing Transcatheter Aortic Valve Replacement. Eur. Radiol..

[10] Michail M., Ihdayhid A.R., Comella A., Thakur U., Cameron J.D., McCormick L.M., Gooley R.P., Nicholls S.J., Mathur A., Hughes A.D. (2021). Feasibility and Validity of Computed Tomography-Derived Fractional Flow Reserve in Patients With Severe Aortic Stenosis: The CAST-FFR Study. Circ. Cardiovasc. Interv..

[11] Peper J., Becker L.M., van den Berg H., Bor W.L., Brouwer J., Nijenhuis V.J., van Ginkel D.J., Rensing B.J.M.W., ten Berg J.M., Timmers L. (2022). Diagnostic Performance of CCTA and CT-FFR for the Detection of CAD in TAVR Work-Up. JACC Cardiovasc. Interv..

[12] Wienemann H., Langenbach M.C., Mauri V., Banazadeh M., Klein K., Hohmann C., Lee S., Breidert I., Hof A., Eghbalzadeh K. (2022). Feasibility and Comparison of Resting Full-Cycle Ratio and Computed Tomography Fractional Flow Reserve in Patients with Severe Aortic Valve Stenosis. J. Cardiovasc. Dev. Dis..

[13] Nørgaard B.L., Fairbairn T.A., Safian R.D., Rabbat M.G., Ko B., Jensen J.M., Nieman K., Chinnaiyan K.M., Sand N.P., Matsuo H. (2019). Coronary CT Angiography-Derived Fractional Flow Reserve Testing in Patients with Stable Coronary Artery Disease: Recommendations on Interpretation and Reporting. Radiol. Cardiothorac. Imaging.

[14] Gohmann R.F., Seitz P., Pawelka K., Majunke N., Schug A., Heiser L., Renatus K., Desch S., Lauten P., Holzhey D. (2022). Clinical Medicine Combined Coronary CT-Angiography and TAVI Planning: Utility of CT-FFR in Patients with Morphologically Ruled-Out Obstructive Coronary Artery Disease. J. Clin. Med..

[15] De Bruyne B., Hersbach F., Pijls N.H.J., Bartunek J., Bech J.W., Heyndrickx G.R., Gould K.L., Wijns W. (2001). Abnormal Epicardial Coronary Resistance in Patients With Diffuse Atherosclerosis but “Normal” Coronary Angiography. Circulation.

[16] Leipsic J., Abbara S., Achenbach S., Cury R., Earls J.P., Mancini G.J., Nieman K., Pontone G., Raff G.L. (2014). SCCT Guidelines for the Interpretation and Reporting of Coronary CT Angiography: A Report of the Society of Cardiovascular Computed Tomography Guidelines Committee. J. Cardiovasc. Comput. Tomogr..

[17] Gohmann R.F., Schug A., Pawelka K., Seitz P., Majunke N., El Hadi H., Heiser L., Renatus K., Desch S., Leontyev S. (2023). Interrater Variability of ML-Based CT-FFR during TAVR-Planning: Influence of Image Quality and Coronary Artery Calcifications. Front. Cardiovasc. Med..

[18] Chinnaiyan K.M., Akasaka T., Amano T., Bax J.J., Blanke P., De Bruyne B., Kawasaki T., Leipsic J., Matsuo H., Morino Y. (2017). Rationale, Design and Goals of the HeartFlow Assessing Diagnostic Value of Non-Invasive FFRCT in Coronary Care (ADVANCE) Registry. J. Cardiovasc. Comput. Tomogr..

[19] Kottner J., Audigé L., Brorson S., Donner A., Gajewski B.J., Hróbjartsson A., Roberts C., Shoukri M., Streiner D.L. (2011). Guidelines for Reporting Reliability and Agreement Studies (GRRAS) Were Proposed. J. Clin. Epidemiol..

[20] Shrout P.E., Fleiss J.L. (1979). Intraclass Correlations: Uses in Assessing Rater Reliability. Psychol. Bull..

[21] Cicchetti D.V. (1994). Guidelines, Criteria, and Rules of Thumb for Evaluating Normed and Standardized Assessment Instruments in Psychology. Psychol. Assess..

[22] Gaur S., Bezerra H.G., Lassen J.F., Christiansen E.H., Tanaka K., Jensen J.M., Oldroyd K.G., Leipsic J., Achenbach S., Kaltoft A.K. (2014). Fractional Flow Reserve Derived from Coronary CT Angiography: Variation of Repeated Analyses. J. Cardiovasc. Comput. Tomogr..

[23] Yang D.H., Kim Y.H., Roh J.H., Kang J.W., Ahn J.M., Kweon J., Lee J.B., Choi S.H., Shin E.S., Park D.W. (2017). Diagnostic Performance of On-Site CT-Derived Fractional Flow Reserve versus CT Perfusion. Eur. Heart J. Cardiovasc. Imaging.

[24] Donnelly P.M., Kolossváry M., Karády J., Ball P.A., Kelly S., Fitzsimons D., Spence M.S., Celeng C., Horváth T., Szilveszter B. (2018). Experience With an On-Site Coronary Computed Tomography-Derived Fractional Flow Reserve Algorithm for the Assessment of Intermediate Coronary Stenoses. Am. J. Cardiol..

[25] Giannopoulos A.A., Keller L., Sepulcri D., Boehm R., Garefa C., Venugopal P., Mitra J., Ghose S., Deak P., Pack J.D. (2023). High-Speed On-Site Deep Learning-Based FFR-CT Algorithm: Evaluation Using Invasive Angiography as the Reference Standard. AJR Am. J. Roentgenol..

[26] Ihdayhid A.R., Sakaguchi T., Kerrisk B., Hislop-Jambrich J., Fujisawa Y., Nerlekar N., Cameron J.D., Seneviratne S.K., Ko B.S. (2020). Influence of Operator Expertise and Coronary Luminal Segmentation Technique on Diagnostic Performance, Precision and Reproducibility of Reduced-Order CT-Derived Fractional Flow Reserve Technique. J. Cardiovasc. Comput. Tomogr..

[27] Kumamaru K.K., Angel E., Sommer K.N., Iyer V., Wilson M.F., Agrawal N., Bhardwaj A., Kattel S.B., Kondziela S., Malhotra S. (2019). Inter- and Intraoperator Variability in Measurement of On-Site CT-Derived Fractional Flow Reserve Based on Structural and Fluid Analysis: A Comprehensive Analysis. Radiol. Cardiothorac. Imaging.

[28] Nicol E.D., Stirrup J., Roughton M., Padley S.P.G., Rubens M.B. (2009). 64-Channel Cardiac Computed Tomography: Intraobserver and Interobserver Variability (Part 1): Coronary Angiography. J. Comput. Assist. Tomogr..

[29] Kerl J.M., Schoepf U.J., Bauer R.W., Tekin T., Costello P., Vogl T.J., Herzog C. (2012). 64-Slice Multidetector-Row Computed Tomography in the Diagnosis of Coronary Artery Disease: Interobserver Agreement among Radiologists with Varied Levels of Experience on a per-Patient and per-Segment Basis. J. Thorac. Imaging.

[30] Murphy M.L., Galbraith J.E., de Soyza N. (1979). The Reliability of Coronary Angiogram Interpretation: An Angiographic-Pathologic Correlation with a Comparison of Radiographic Views. Am. Heart J..

[31] Cook C.M., Petraco R., Shun-Shin M.J., Ahmad Y., Nijjer S., Al-Lamee R., Kikuta Y., Shiono Y., Mayet J., Francis D.P. (2017). Diagnostic Accuracy of Computed Tomography-Derived Fractional Flow Reserve: A Systematic Review. JAMA Cardiol..

[32] Tanigaki T., Emori H., Kawase Y., Kubo T., Omori H., Shiono Y., Sobue Y., Shimamura K., Hirata T., Matsuo Y. (2019). QFR Versus FFR Derived From Computed Tomography for Functional Assessment of Coronary Artery Stenosis. JACC Cardiovasc. Interv..

[33] Rajiah P., Cummings K.W., Williamson E., Young P.M. (2022). CT Fractional Flow Reserve: A Practical Guide to Application, Interpretation, and Problem Solving. Radiographics.

[34] Tsugu T., Tanaka K., Belsack D., Nagatomo Y., Tsugu M., Argacha J.F., Cosyns B., Buls N., De Maeseneer M., De Mey J. (2024). Impact of Vessel Morphology on CT-Derived Fractional-Flow-Reserve in Non-Obstructive Coronary Artery Disease in Right Coronary Artery. Eur. Radiol..

[35] Van Hamersvelt R.W., Voskuil M., De Jong P.A., Willemink M.J., Išgum I., Leiner T. (2019). Diagnostic Performance of On-Site Coronary CT Angiography–Derived Fractional Flow Reserve Based on Patient-Specific Lumped Parameter Models. Radiol. Cardiothorac. Imaging.

[36] Yan H., Gao Y., Zhao N., Geng W., Hou Z., An Y., Zhang J., Lu B. (2022). Change in Computed Tomography-Derived Fractional Flow Reserve Across the Lesion Improve the Diagnostic Performance of Functional Coronary Stenosis. Front. Cardiovasc. Med..

[37] Cami E., Tagami T., Raff G., Fonte T.A., Renard B., Gallagher M.J., Chinnaiyan K., Bilolikar A., Fan A., Hafeez A. (2018). Assessment of Lesion-Specific Ischemia Using Fractional Flow Reserve (FFR) Profiles Derived from Coronary Computed Tomography Angiography (FFRCT) and Invasive Pressure Measurements (FFRINV): Importance of the Site of Measurement and Implications for Patient Referral for Invasive Coronary Angiography and Percutaneous Coronary Intervention. J. Cardiovasc. Comput. Tomogr..

[38] Chen M., Almeida S.O., Sayre J.W., Karlsberg R.P., Packard R.R.S. (2024). Distal-Vessel Fractional Flow Reserve by Computed Tomography to Monitor Epicardial Coronary Artery Disease. Eur. Heart J. Cardiovasc. Imaging.

[39] Renker M., Baumann S., Hamm C.W., Tesche C., Kim W.K., Savage R.H., Coenen A., Nieman K., De Geer J., Persson A. (2021). Influence of Coronary Stenosis Location on Diagnostic Performance of Machine Learning-Based Fractional Flow Reserve from CT Angiography. J. Cardiovasc. Comput. Tomogr..

[40] Knuuti J., Wijns W., Saraste A., Capodanno D., Barbato E., Funck-Brentano C., Prescott E., Storey R.F., Deaton C., Cuisset T. (2020). 2019 ESC Guidelines for the Diagnosis and Management of Chronic Coronary Syndromes: The Task Force for the Diagnosis and Management of Chronic Coronary Syndromes of the European Society of Cardiology (ESC). Eur. Heart J..

[41] Tesche C., Otani K., De Cecco C.N., Coenen A., De Geer J., Kruk M., Kim Y.H., Albrecht M.H., Baumann S., Renker M. (2020). Influence of Coronary Calcium on Diagnostic Performance of Machine Learning CT-FFR: Results From MACHINE Registry. JACC Cardiovasc. Imaging.

